# Endosomal accumulation of APP in wobbler motor neurons reflects impaired vesicle trafficking: Implications for human motor neuron disease

**DOI:** 10.1186/1471-2202-12-24

**Published:** 2011-03-07

**Authors:** Ralf Palmisano, Panagiota Golfi, Peter Heimann, Christopher Shaw, Claire Troakes, Thomas Schmitt-John, Jörg W Bartsch

**Affiliations:** 1 King's College London, Pharmaceutical Science Research Division, 150 Stamford Street, London SE1 9NH, UK; 2 Department of Cell Biology, Bielefeld University, 33501 Bielefeld, Germany; 3 Kings College London, Institute of Psychiatry, De Crespigny Park, London SE5 8AF, UK; 4 Molecular Biology Department, Aarhus University, 8000 Aarhus, Denmark; 5 Department of Neurosurgery, University of Marburg, Baldingerstrasse, 35033 Marburg, Germany

## Abstract

**Background:**

The cause of sporadic amyotrophic lateral sclerosis (ALS) is largely unknown but hypotheses about disease mechanisms include oxidative stress, defective axonal transport, mitochondrial dysfunction and disrupted RNA processing. Whereas familial ALS is well represented by transgenic mutant SOD1 mouse models, the mouse mutant wobbler (WR) develops progressive motor neuron degeneration due to a point mutation in the *Vps54 *gene, and provides an animal model for sporadic ALS. VPS54 protein as a component of a protein complex is involved in vesicular Golgi trafficking; impaired vesicle trafficking might also be mechanistic in the pathogenesis of human ALS.

**Results:**

In motor neurons of homozygous symptomatic WR mice, a massive number of endosomal vesicles significantly enlarged (up to 3 μm in diameter) were subjected to ultrastructural analysis and immunohistochemistry for the endosome-specific small GTPase protein Rab7 and for amyloid precursor protein (APP). Enlarged vesicles were neither detected in heterozygous WR nor in transgenic SOD1(G93A) mice; in WR motor neurons, numerous APP/Rab7-positive vesicles were observed which were mostly LC3-negative, suggesting they are not autophagosomes.

**Conclusions:**

We conclude that endosomal APP/Rab7 staining reflects impaired vesicle trafficking in WR mouse motor neurons. Based on these findings human ALS tissues were analysed for APP in enlarged vesicles and were detected in spinal cord motor neurons in six out of fourteen sporadic ALS cases. These enlarged vesicles were not detected in any of the familial ALS cases. Thus our study provides the first evidence for wobbler-like aetiologies in human ALS and suggests that the genes encoding proteins involved in vesicle trafficking should be screened for pathogenic mutations.

## Background

Neurodegenerative processes cause dramatic but heterogeneous disease phenotypes depending on the onset of symptoms, disease progression and the particular type of neurons being affected. In the case of "Amyotrophic Lateral Sclerosis" (ALS) the motor neurons of the motor cortex, brain stem and spinal cord are affected. The degeneration of motor nerves causes denervation of skeletal muscle and progressive muscular weakness leading eventually to paralysis and death. Despite intensive research no effective therapeutic treatment is available but recently some progress has been made in the understanding of the underlying molecular mechanisms of ALS.

The majority of neurodegenerative disorders are associated with abnormal protein aggregation. Aggregates of amyloidogenic cleavage products of amyloid precursor protein (APP) are implicated in the pathogenesis of Alzheimer disease (AD, recently reviewed by [1]). APP accumulation also occurs within intracellular vesicles in Niemann Pick Disease type C (NPC) [2] and recently found to be elevated in skeletal muscles of ALS patients as well as SOD1-G93A mutant transgenic mouse [3]. For these reasons we elected to investigate APP accumulation in the wobbler mouse, another animal model for human ALS with a different pathomechanism.

The recessive *wobbler *mutation (*wr *= gene symbol, phenotype WR) spontaneously occurred 50 years ago in the breeding stock of Falconer [4] and was later mapped to the proximal mouse chromosome 11 [5]. Homozygous (*wr/wr*) wobbler mice develop the first disease symptoms at the age of three to four weeks. From this time point onwards the muscle weakness, beginning in the forelegs, proceeds to death. The life expectancy of homozygous WR mice is around 120 days. Degeneration of WR motor neurons is accompanied by activation of glia cells (reactive gliosis and microglia activation) and shows striking similarities to early-onset ALS cases. The phenotype of the wobbler mouse has been intensively investigated throughout the last 50 years and studies towards potential therapies have been conducted. A positional cloning of the *wobbler *gene revealed a highly conserved vesicle trafficking factor, Vps54 [6]. The *wobbler *mutation is a point mutation leading to an amino acid replacement (Q967L) in the C-terminal domain of Vps54 causing the ALS-like motor neuron degeneration. We also demonstrated that the complete loss of Vps54 function leads to embryonic lethality around day 11.5 of embryonic development [6].

Yeast, as well as mammalian Vps54 forms a complex with Vps52 and Vps53, called GARP (Golgi associated retrograde protein) complex [7,8], which is required for tethering and fusion of endosome-derived transport vesicles to the trans-Golgi network (TGN) [9]. The GARP complex tethers vesicles to their target membrane (TGN) leading to a t-SNARE - v-SNARE dependent fusion of the membranes. In mammalian cells, Vps52 interacts with the tSNARE Syntaxin10 and the small GTP binding protein Rab6 [8]. In wobbler mice, mutation of leucine-967 to glutamine causes instability of an alpha helical structure, leading to reduced levels of Vps54 and consequently, the GARP complex [9]. In this study we characterise abnormally enlarged endosomal vesicles in wobbler spinal cord motor neurons and provide evidence that similar vesicles can be found in motor neurons of a subset of human ALS cases. These findings provide evidence for morphological criteria that could be used to classify human MND.

## Results

### Fine structure of WR motor neurons

**Figure 1:**
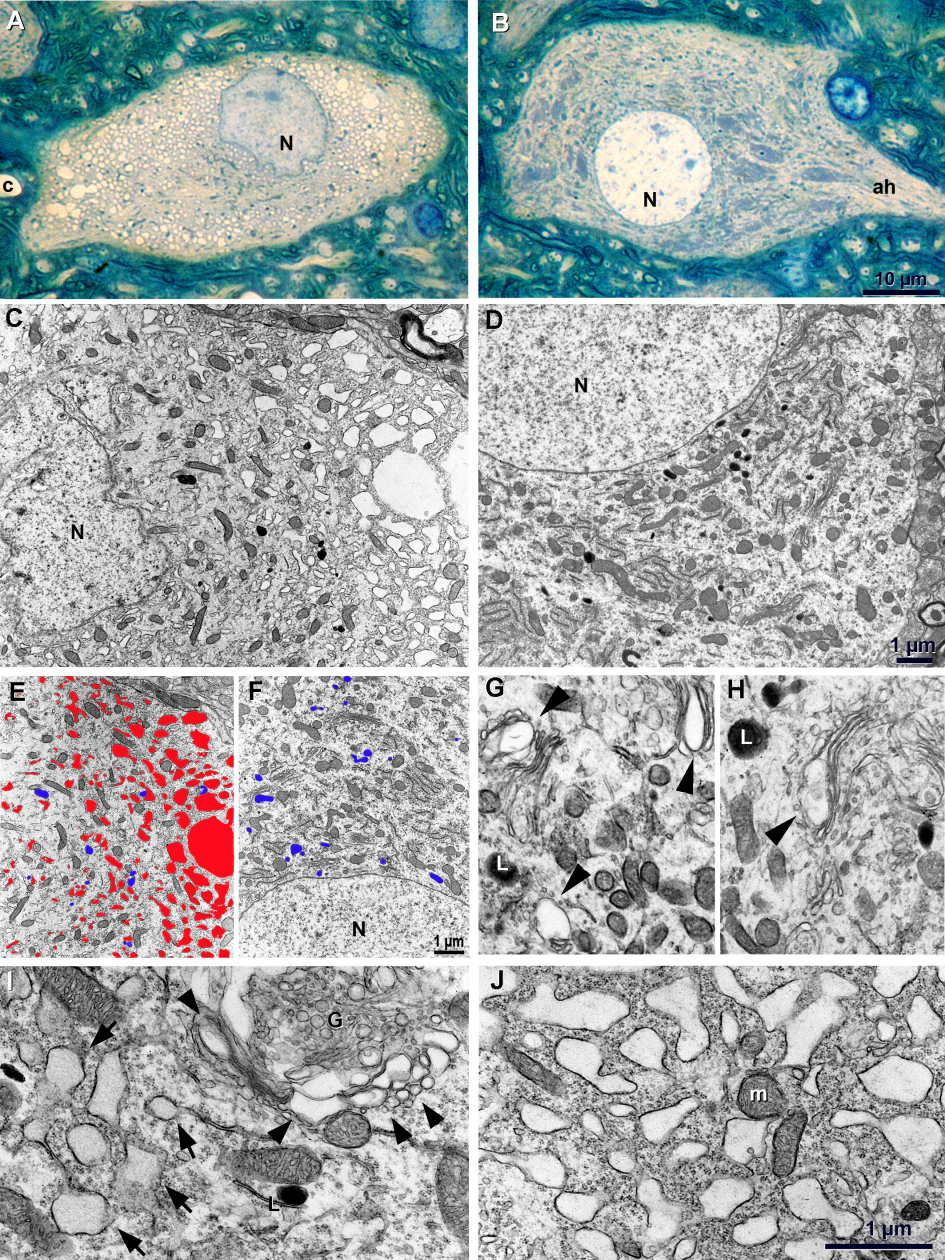
**Fine structure of wobbler and WT motor neurons**. (A) Terminal stage of WR motor neuron. Note extensive vacuolisation but normal shape of cell bodies and non-necrotic and non-apoptotic nucleus (N). (B) Intact WR motor neuron from contralateral side of same section as in (a) with Nissl-Golgi areas (darker blue areas) within the cell body. (C) Terminal stage of motor neuron death in WR is characterized by extensive vacuolisation but otherwise intact organelle structure, frequency and distribution. (D) Motor neuron from age-matched WT, lysosomes discernible (as in C) as deep-black organelles. (E, F) Camera lucida sketch of wr terminal stage (E) and of intact motor neuron from wildtype (F); vacuoles highlighted in red, lysosomes in blue. (G, H) Earliest changes are observed by clear enlarged vesicles / vacuoles (arrowhead) derived from Golgi apparatus. (I) Later stage of motor neuron death is characterized by massive vacuolisation of Golgi apparatus (arrowheads) and additionally by vacuolisation/dilatation of ER with grey electron dense content (cytoplasm; arrows). (J) Final stage of motor neuron death with numerous and large vacuoles but mainly intact mitochondria. N = nucleus; c = capillary; ah = axon hillock, m = mitochondrion. Scale in B (for A &B), 10 μm; in D (for C & D), in F (for E & F), and J (G-J), 1 μm, respectively.

To analyse the consequence of impaired vesicle trafficking in WR motor neurons, light (Figure 1A&B) and electron microscopy (EM, Figure 1C-J) of spinal cord sections from symptomatic wobbler (42 d.p.n.) and age-matched unaffected controls (heterozygous *wr/+*) was performed. In wobbler spinal cord, a small fraction of motor neurons is symptomatic for neurodegeneration, judged by morphological changes ranging from early vacuolisation to obvious neuronal cell death (Figure 1A&1B). Degeneration stages were classified based on Nissl staining as described earlier [10]. Though nuclear morphology changes from distinct rounded contours to slightly irregular ones (Figure 1A&1C), no change in nuclear morphology, as characteristic for apoptosis or necrosis can be observed, even at later stages of degeneration. Changes at the ultra structural level are transparent enlarged vesicles / vacuoles which are derived from the Golgi apparatus (Figure 1G&1H). In more advanced stages of degeneration, these vacuoles increase in size and become more frequent (Figure 1I). A second type of vacuoles, according to their position and their association with ribosomes, appear to originate from dilatation of the endoplasmic reticulum (ER), as demonstrated in earlier stages (postnatal day 7 to 14) of the wobbler disease [11]. At terminal stages, motor neuron degeneration is characterized by extensive vacuolisation (Figure 1A, C, E, J) with vacuoles graded from smaller up to extremely large (2-3 μm diameter) ones (Figure 1C, E, J). Initially, areas with strong vacuolisation seem to be localized in one region of the cell (Figure 1C, E) but later they are found all over the motor neuronal soma (Figure 1A) obviously sparing the axon and the axon hillock. No difference in number and distribution of lysosomes was observed between wobbler and wild-type motor neurons (Figure 1C, D, E, F).

### Rab7 and APP colocalise in enlarged endosomal structures in wobbler spinal cord motor neurons

**Figure 2:**
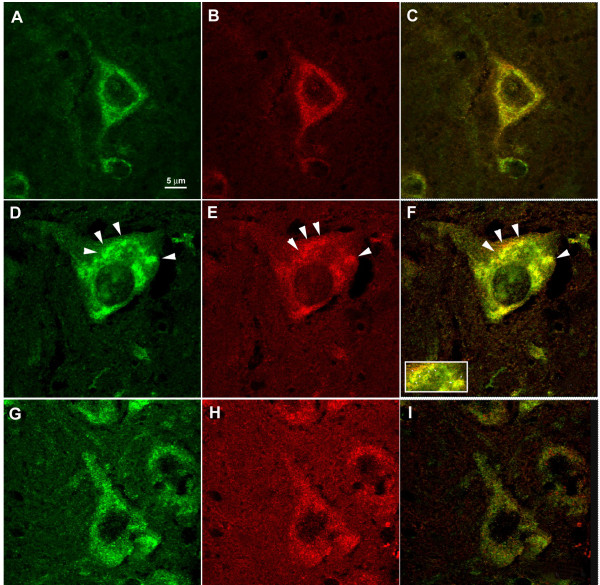
**APP and Rab7 colocalise in degenerating motor neurons**. Representative immunohistochemical staining for Amyloid-Precursor Protein (APP) (A,D,G) using rabbit anti-APP, and for Rab7 (B,E,H) using goat-anti-Rab7, and co-localisation of both proteins (C,F,I) in spinal cord motor neurons of 42 d.p.n. wild-type (WT, A,B,C), age-matched wobbler (WR, D,E,F), and 120 d.p.n. SOD1G93A (G,H,I) transgenic mice. Note the spotty appearance of the large vesicular structures with up to 2.5 μm diameter in WR sections. These structures were not seen in WT and in SOD1 (G93A) transgenic spinal cord sections. Bar in A, valid for A-I, 5 μm.

**Figure 3:**
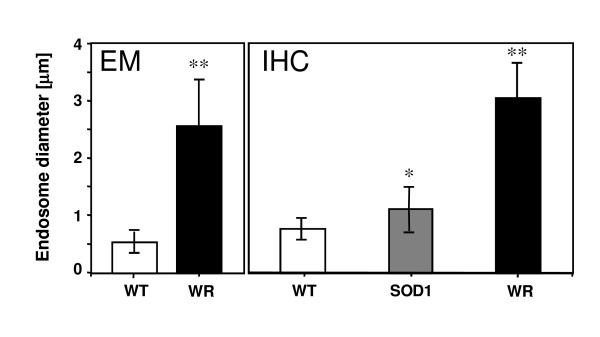
**Enlarged vesicles in WR motor neurons**. Quantification of vesicle diameters in WT, WR and SOD1 (G93A) tg motor neurons, judged by electron microscopy (WT, WR) and by immunohistochemistry (WT, WR, SOD1 (G93A)tg). Cross-sectional vesicle diameters were determined by ImageJ analysis (see M&M section) for 100 different APP-positive motor neurons in serial cervical spinal cord sections (n = 10 per genotype, 10 sections per individual) from WR, WT, and SOD1 (G93A) tg mice, respectively. Values are given as mean values ± SD. P-values < 0.01 between WT, WR, and SOD1(G93A) were considered significant with *, p < 0.01 and **, p < 0.001.

As demonstrated earlier, the small GTPase protein Rab7 has a role in late endosomal pathway and in the final maturation of autophagic vesicles [12]. The wobbler mutation causes impaired retrograde transport of vesicles. Thus we speculated that Rab7 might be expressed differently in wobbler motor neurons. Spinal cord sections from unaffected heterozygous (genotype *wr/+*) and wobbler (WR, genotype *wr/wr*) mice were stained for Rab7 (Figure 2B, E) and revealed enlarged vesicles in wobbler motor neurons compared to wild-type controls. Interestingly, staining for APP, an abundant membrane protein, which is transported via the secretory pathway, showed an identical localisation as Rab7 preferentially in endosomes (Figure 2A, D), indicating that APP and Rab7 are co-localised in enlarged vesicles in wobbler motor neurons (Figure 2F). This type of co-localisation was only seen in degenerating motor neurons, whereas morphologically normal motor neurons showed no such abnormalities. In addition, spinal cord motor neurons from symptomatic SOD1 tg (G93A) mice (120 d.p.n.) exert a vesicular distribution of APP and Rab7 distinct from the observations made in WR motor neurons (Figure 2G, H, J). The enlarged vesicles characterised by EM and by APP immunohistochemistry (IHC) were subjected to image analysis to quantify cross-sectional vesicular diameters in wildtype, wobbler, and SOD1 tg (G93A) mice (Figure 3). A significant difference was seen in endosomal diameters in wild-type and wobbler motor neurons, both by EM and by IHC to a similar extent (0.5 μm vs. 2.5 μm, and 0.8 vs. 3 μm, respectively), whereas the endosomes in SOD1 tg (G93A) motor neurons were only slightly enlarged (1 μm vs 0.8 μm in WT, p < 0.001), supporting the notion that large endosomal aggregates are indicative for the WR disease with impaired vesicle trafficking.

### Distinct localisation of APP and LC3 in degenerating motor neurons

**Figure 4:**
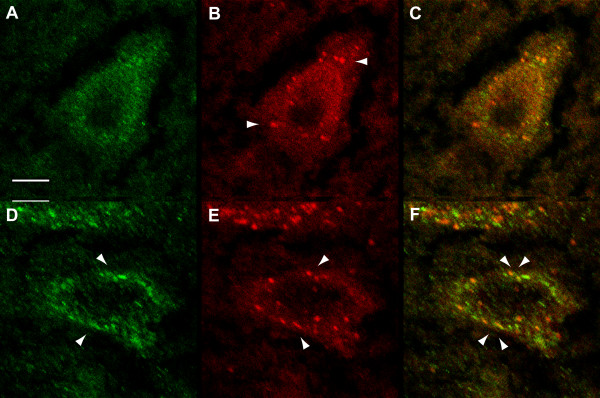
**APP/LC3 immunohistochemistry in WR motor neurons**. Immunostaining for APP (A,D; green) using rabbit-anti-APP and mouse-anti-LC3 (B,E; red) performed in either WT (A,B) or WR (D,E) motor neurons in cervical spinal cord (42 d.p.n.). Note a similar number of autophagosomes in B and E (arrowheads). Arrows in D indicates large APP-positive vesicles. Merged images in C and F demonstrate that APP and LC3 are localised to distinct vesicles in degenerating motor neurons. Bar in A, valid for A-E, 10 μm.

To test the hypothesis that enlarged vesicles in WR motor neurons could be autophagosomes, co-stains of APP and LC3 were performed in a representative motor neuron with normal (WT, Figure 4A, B, C) and with degenerating morphology (WR, Figure 4D, E, F). In both motor neuron populations, LC3-positive vesicles were indistinguishable in size and number between WT and WR, and most of APP-positive large vesicles in wobbler motor neurons did not stain for LC3 and vice versa (Figure 4C, F). We conclude that the abnormally enlarged vesicles in WR motor neurons are distinct from autophagosomes.

### Analysis of APP processing in brain stem and spinal cord of wild-type and wobbler mice

**Figure 5:**
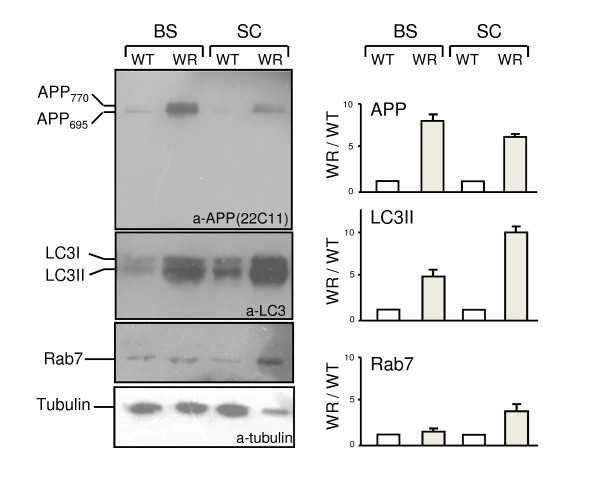
**APP processing in WR spinal cord**. Western blot analysis of brain stem (BS) and spinal cord (SC) lysates from age-matched WT and WR mice. Total lysates (20 μg per lane) were separated on an 8% polyacrylamide gel, blotted and subjected to immunostaining using 22C11 (APP), LC3 and Rab7 as described. The positions of APP695 and APP770, of LC3I (18kD form) and LC3II (16 kD form) and Rab7 (22 kD) are marked on the left. Anti-tubulin antibody (lower panel) was used as loading control. Band intensities for APP695, LC3II and Rab7 were quantified from 3 independent experiments and plotted as ratio WR/WT ± SEM. For each WT/WR pair, the WT value was set to 1.

Accumulation of APP could be due to abnormal secretase processing. Due to a distinct expression of APP in motor neurons in the spinal cord (see Figure 2) we analysed brain stem and spinal cord protein extracts from WT and WR mice for expression levels of APP, LC3 and Rab7 (Figure 5). Significant increases in APP, LC3 and Rab7 protein levels were detected in WR brain stem and spinal cord lysates compared to WT. No APP processing products, however, were detected, suggesting that accumulation of APP is not due to abnormal processing in WR motor neurons. The levels of LC3 expression is higher in WR spinal cord, however the ratios of LC3I (18kD) and LC3II (16kD) were unchanged, indicating that autophagosome activation is not affected. For Rab7 protein, a slight increase in WR spinal cord was also observed, concomitant with the observation of endosome accumulation in WR motor neurons (Figure 2). With respect to APP processing, we stained WT and WR spinal cord sections for the secretases ADAM10 and BACE, but neither a difference in distribution nor in concentration was detected (data not shown).

### APP-positive enlarged vesicles in human MND/ALS autopsies

**Figure 6:**
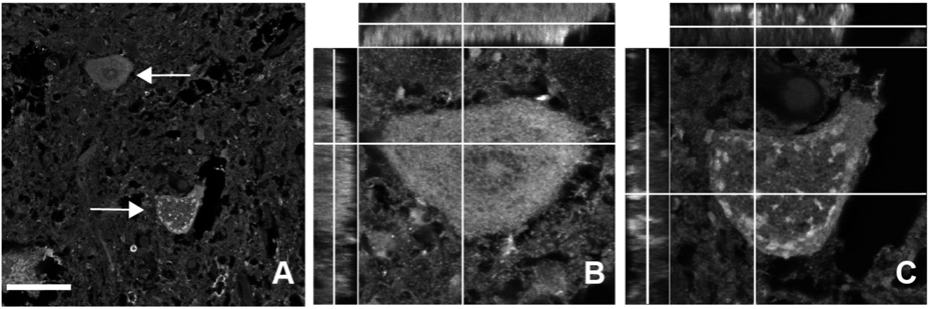
**APP staining in the spinal cord of human ALS autopsies**. Representative (case No. 12 see table 1 for details) degenerating human motor neuron with enlarged APP-positive vesicles in the cell soma. For comparison, a morphologically normal spinal cord motor neuron from the same autopsy is shown above (arrow). (A), a single plane image, (B) and (C), maximum projections from a stack of images obtained from confocal optical sectioning. (A), low magnification of MND spinal cord section stained with anti-APP. (B), high power magnification of the normal MN shown in (A), as a xy- (middle), yz- (left) and xz- (top) scan with diffuse staining of APP in the cell soma; (C), high power magnification of the degenerating MN shown in A, as an xy- (middle), yz- (left) and xz- (top) scan with enlarged vesicles, some of them larger than 2 μm (see crossing point of the planes in C with a vesicle diameter of 2.8 μm. The yz- and xz-scans reveal the three-dimensional APP-positive vesicles in the degenerating human motor neuron. Scale bar in (A), 50 μm.

From these previous results we concluded that the presence of enlarged APP-positive vesicles could be indicative for motor neuron diseases with impaired retrograde protein transport. We analysed a collection of human MND autopsy samples with well-documented case histories but unknown aetiologies for the presence of APP positive enlarged vesicles after immunohistochemistry (Figure 6 and Table 1). From fourteen documented MND cases investigated, six MND cases displayed enlarged vesicles in spinal cord motor neurons. Two familial MND cases with mutations in the *SOD *gene did not exert enlarged vesicles, which could match our findings in SOD1(G93A) motor neurons and implies that SOD1 mutations are distinct in motor neuron degeneration mechanisms from the sporadic ALS cases with impaired trafficking that we investigated here. Motor neurons with enlarged vesicles were found in individual motor neurons in the spinal cord of autopsies with ALS or Motor neurons disease (MND), a representative example of this staining is shown (Figure 6). Analyses of APP immunoreactivity in MND patients in optical z- and y-axes revealed that APP is present in vesicle structures with diameters of 1.5-4 μm (average 2.8 μm), very similar to the dimensions of enlarged APP-positive vesicles observed in WR motor neurons.

**Table 1:** APP^+ ^vesicle formation in MND autopsies

CASE #	AGE	GENDER	PM [h]	PATHOLOGICAL DIAGNOSIS	DEG.	APP+ VESICLES	VPS54 SEQ. ALTERATION
**1**	74	M	83	MND	-3	+	n.d.
**2**	49	M	32	MND	-3	-	None
**3**	58	M	37	MND	-2	-	n.d.
**4**	69	F	17	MND	-3	+	n.d.
**5**	48	M	25	MND	-2	+	None
**6**	81	F	30	MND	-2	-	None
**7**	55	M	39	MND	-2	-	None
**8**	72	F	6	MND	-1	-	n.d.
**9**	68	F	70	MND	-3	+	None
**10**	54	M	30	MND	-1	-	n.d.
**11**	51	F	24	MND	-3	+	None
**12**	54	M	95	MND	-2	+	n.d.
**13**	54	F	?	MND Familial	-3	-	n.d.
**14**	56	M	79	MND Familial	-2	-	n.d.
**15**	89	F	54	PD	-1	-	None
**16**	85	F	9	PD	-2	-	None
**17**	61	M	65	Amyloid plaques	0	-	n.d.
**18**	79	M	24	Mild ageing changes	0	-	n.d.
**19**	80	M	48	changes consistent with ageing	0	-	n.d.
**20**	40	M	40	Normal adult brain	0	-	n.d.
**21**	57	M	26	Normal adult brain	0	-	n.d.
**22**	87	F	21	Normal adult brain	0	-	None
**23**	68	M	53	Normal adult brain	0	-	n.d.
**24**	64	F	60	Normal adult brain	0	-	n.d.
**25**	61	M	35	Normal adult brain	0	-	n.d.
**26**	58	F	21	Mild ischemia	0	-	None
**27**	68	M	40	Diffuse hypoxic damage	0	-	n.d.
**28**	61	M	65	None given	0	-	n.d.

Legend: Histological assessment of human Motor neuron disease (MND) autopsies from spinal cord. Spinal cord sections were fixed in formalin after the post-mortem time indicated ("PM", in hours [h]) and later embedded in paraffin. The grades of Motor neuron degeneration were judged by Nissl staining and designated as 0 (no degeneration), -1 (single degenerating neurons), -2 (more than one degenerating motor neuron per ventral horn), -3 (groups of degenerating motor neurons). Note that in non-ALS brains including normal adult brain or other origins of neurodegeneration, APP^+ ^vesicles have not been detected.

### Sequence analysis of Vps54 gene of ALS patients

Genomic DNAs of all six ALS patients with wobbler-like enlarged endosomal structures as well as controls were prepared from cerebellum samples. All exons of human VPS54 were amplified including flanking regions, and sequenced in both orientations. In none of the samples and none of the VPS54 exons a sequence variation could be detected, thus, no mutation or polymorphism could be found in the VPS54 gene of the selected ALS patients.

## Discussion

Our study demonstrates that impaired retrograde transport as observed in wobbler motor neurons is characterized by the presence of enlarged endosomal vesicles with positive immunostaining for Rab7 and APP, but not for LC3. As such, the type of motor neuron degeneration as observed in WR does not involve autophagosomes. These findings contrast those for motor neurons of SOD1(G93A) mice where extensive autophagosome activation has been demonstrated [13]. It is unlikely that the vesicular accumulation of APP is instrumental for motor neuron degeneration in the wobbler mouse, as APP transgenic mice crossed into the wobbler mutation did not enhance motor neuron pathology (Schmitt-John et al, unpublished observations).

Characteristic signs of motor neuron death in wobbler are enlarged vesicles blebbing of the Golgi apparatus, increasing in size and number in later stage as well as enlarged vesicles/vacuoles derived from the ER, in agreement with earlier EM studies performed on wobbler motor neurons [11]. With the exception of this vacuolisation, the affected motor neuron seems to be viable just up to the terminal breakdown. The ultrastructural data indicate that the vesicular shuttle system of ER and Golgi and perhaps of lysosomes (GERL-system) is affected. This would be in agreement with the fact that in wobbler mice the vesicular transport factor VPS54, a component of the GARP complex, is mutated [6]. In particular, vacuolisation in close proximity to the Golgi apparatus seen on electron micrographs of wobbler motor neurons reflects the function of the GARP complex involved in tethering endosome-derived retrograde vesicles to the Golgi membrane as shown in yeast [7], but also in mammalian cells [14].

Immunofluorescence data indicate an intracellular accumulation of APP in Rab7- but not LC3-positive endosomal compartments in degenerating wobbler motor neurons. Intracellular APP accumulations have earlier been reported for Niemann Pick Disease type 1 (NPC1), where APP was found in Rab5-positive early endosomes rather than in Rab7 positive late endosomes [2]. Furthermore, Jin et al. [2] found elevated, proteolytically processed APP (C99 and Aβ42) in NPC1 neurons. The western blotting data suggest that in wobbler spinal cords APP is upregulated but no indications for abnormal APP proteolysis have been found indicating potential amyloid accumulations. Thus, we conclude that the wobbler vesicle trafficking defect leads to an accumulation of native APP variants in abnormally enlarged APP^+^/Rab7^+ ^endosomal compartments.

The average cross-sectional diameter of these compartments is 3 μm and significantly larger than APP- and Rab7-positive structures in wild-type or SOD1 transgenic mice. The ultrastructural equivalents are vacuoles with low electron density and a size of up to 3 μm. These vacuoles most likely are derived from the Golgi and the ER. Since the occurrence of such structures is found in wobbler, but not in SOD1 and wild type mice these are likely caused by impaired bidirectional vesicle traffic between ER and Golgi. Again, these findings are in agreement with the function of Vps54 and the GARP complex as recently demonstrated [14].

Enlarged APP^+^/Rab7^+ ^endosomes were also found in MDF ("muscle deficient") spinal cord motor neurons. Recently, the mutation underlying the mouse mutant MDF has been revealed as scyl1, a gene that affects Golgi transport and morphology [15] (see additional file 1), confirming that vesicular APP/Rab7 staining indeed reflects transport impairment in motor neurons.

Extending our analysis to human MND were able to identify APP^+^/Rab7^+ ^vesicles in degenerating motor neurons, which could indicate that in a subset of human MND, retrograde vesicle trafficking is affected and could lead to the identification of more MND relevant gene mutations. Thus the correlation between vesicle trafficking defects and ALS is obvious since the affected gene for familial ALS2 Alsin was found to encode a guanine-nucleotide exchange factor for the vesicle traffic associated protein Rab5 [16,17] and ALS8 encodes the vesicle associated membrane protein B (VAPB, [18]). Besides these, several other vesicle trafficking proteins have been associated with neurodegeneration like Dynactin with Neuropathy distal hereditary motor type VIIB (HMN7B, [19] and Phosphoinositide phosphatase FIG4 in patients with ALS [20].

The VPS54 genes of some MND patients, preselected for enlarged APP- and LC3-positive endosomal compartments were sequenced, but so far, mutations or polymorphisms were not detected. A similar sequencing effort, although without pre-selection for APP storage, has recently been published [21]. In this report a polymorphism in VPS54 has been identified but could not be directly associated with the disease. Thus up to now, mutations or polymorphisms in VPS54 are not defined as a major cause or a risk factor for sporadic forms of MND or ALS.

## Conclusions

Our present study indicates that impaired vesicle trafficking in MND mouse mutants is reflected by APP/Rab7 accumulation in spinal cord motor neurons. Some sporadic forms of human MND or ALS show similar observations, so that assessment of APP localisation in motor neurons could allow classification of human aetiologies with impaired vesicle trafficking in order to identify additional MND-relevant genes.

## Methods

### Mice

Breeding and genotyping of C57Bl/6J-*wr *and C57Bl/6J stock mice was described earlier [6]. The SOD1 G93A transgenic mice were obtained from M. Sendtner (Würzburg University, Germany) and genotyped for the SOD1 (G93A) transgene as described earlier [22]. Animal experimentation was done in accordance with the German law for the protection of animals (TschG) and with a permit by the local authorities.

### Human samples

Human MND and control autopsy samples were derived from the MRC London Neurodegenerative Diseases Brain Bank (Institute of Psychiatry, King's College London) with an ethical approval from local authorities. The case details including post mortem time before formalin fixation or freezing of samples are provided in table 1. Generally, post-mortem time had no effect on the formation of APP-positive vesicles, as spinal cord tissues from wild-type mice were used as controls 120 hours post-mortem time and no changes in vesicular APP was observed (data not shown). Formalin fixed tissues were embedded in paraffin according to standard procedures.

### Immunohistochemistry

Cryosections of mouse CNS tissue (10 μm) were fixed with 3.7% paraformaldehyde in phosphate buffered saline (PBS, 140 mM NaCl, 10 mM Na, K phosphate, pH 7.3). Paraffin sections from human autopsies (5 μm thickness) were deparaffinised using xylene and varying concentrations of ethanol before immunostaining.

For immunohistochemistry, anti-APP directed against the extracellular domain (aa 301-315 of human APP, Sigma A8842, 1:500) was used in conjunction with anti-rabbit-Alexa 488 (1:500, MoBiTec); detection of the endosomal protein Rab7 was performed using goat-anti-Rab7 (SC-6563, Santa Cruz, 1:100) and mouse anti-goat-Cy3 (Dianova, Hamburg, 1:300). Both primary antibodies were used simultaneously in a single-label experiment to avoid cross-reactivity. For autophagosome detection, anti-LC3 antibody (1:200, Nanotools, Munich) was used in conjunction with an anti-mouse Cy3 secondary antibody (1:1000, Sigma) and anti-APP described above in a simultaneous incubation.

### Fluorescence microscopy, confocal microscopy

Confocal images were obtained using Leica SP2 Confocal Laser Scanning Microscope with software version 2.61 build 1537. Images were taken using either HCX PL APO CS 63.0 × 1.32 OIL UV or HC PL APO CS 20.0 × 0.70 IMM/COR objectives. To acquire different fluorescence channels settings were optimized to prevent cross-talk, although images were sequentially scanned to avoid this problem. All images were taken with 4 × line average at data acquisition. Images were merged, and contrast and brightness was enhanced using Leica software. Processing of image stacks was done with Volocity software.

### Electron Microscopy

Mice were anaesthetized and transcardially perfused (with a permit from the local authorities) in a 3 step procedure slightly modified according to Forssmann et al [23] with 3% paraformaldehyde, 3% glutaraldehyde, 0,5% picric acid in 0.1 M sodium phosphate buffer, pH 7.4 for 10 minutes. The spinal cord was dissected and fixed in the same solution for additional 1-2 hours at 4°C, postfixed in 2% osmium tetroxide (2 h, 4°C) and embedded in Araldite resin. For light microscopic identification, sections of 1.5 μm thickness were stained with Richardson's blue (1% w/v methylene blue, 1% w/v Azur II) for 1 min, 80°C and visualised with a 100 × objective at a Zeiss Axiophot microscope. For electron microscopy, 60-80 nm sections (stained for 40 min in uranyl acetate and 7 min in lead citrate) were used (Zeiss EM 109).

### Size determination of endosomal compartment

For quantification of vesicle areas we used the ImageJ program http://rsb.info.nih.gov/ij/download.html. Images were imported into image J, the scale was set to a defined length for normalisation. Vesicles on the images were determined for cross-sectional diameters [μm]. Data were obtained from a total of 100 motor neurons from 10 spinal cord sections per individual (n = 10 per genotype, age-matched 42 d.p.n.) and are presented as mean ± SD. Significance was determined using a paired *t*-test and values of p < 0.01 were considered significant.

### Western Blot analysis

CNS tissues (cervical spinal cord and brain stem) were removed quickly and homogenized in a buffer containing 100 mM Tris-HCl, pH7.4, 1 mM EGTA, and Complete Inhibitor Mix (Boehringer Mannheim). After addition of Laemmli's buffer, the protein extracts were denatured at 100°C for 5 min and separated on a 8% SDS-polyacrylamide gel. Proteins were immobilized by capillary blot on a nitrocellulose membrane (Protran; Schleicher & Schüll, Dassel, Germany). The transfer was checked by reversible ponceau staining, and unspecific binding sites were blocked overnight with blocking buffer [5% nonfat milk powder in TTBS (20 mM Tris-HCl, pH 7.5, 500 mM NaCl, and 0.05% Tween 20]. For detection of Amyloid-Precuror protein APP in western blots, monoclonal antibody 22C11 (Millipore, Watford, UK; 1:1000) was incubated with the membranes for 4 hr at room temperature. Similarly, antibodies directed against LC3 (1:1000) and Rab7 (1:200) were used. After extensive washing (four times for 15 min each) with TTBS-0.5% non-fat milk powder, membranes were incubated with either rat anti-mouse (APP and LC3, 1:10000; Jackson ImmunoResearch, West Grove, PA) or rabbit anti-goat (Rab7, 1:2000) conjugated with horseradish peroxidase, respectively, for 45 min. After washing (three times for 10 min each) with TTBS, protein bands were detected with Lumi-Light Plus Western blotting substrate (Roche Applied Science) and Kodak X-OMAT film (Eastman Kodak, Rochester, NY).

### DNA preparation PCR amplification and sequencing

Genomic DNA from frozen cerebellum samples (500 mg) of selected human patients and controls was prepared by grinding in liquid nitrogen according to standard protocols [24]. All 23 exons of human *VPS54 *gene were PCR amplified from 100 ng genomic DNA using standard protocols, primer sequences and annealing temperatures can be obtained from Thomas Schmitt-John upon request. PCR products were separated on 1% agarose gels, bands excised and the DNA was extracted using QiaQuick gel extraction kit (Qiagen, Hilden). Gel-purified PCR products were subjected to DNA sequencing using either forward or reverse PCR primer, Big Dye 3 cycle sequencing and an ABI 3130 × l sequencing machine (Applied Biosystems).

## Authors' contributions

All authors read and approved the final manuscript. RP performed the confocal microscopy work, PG carried out the Nissl and immunostains and the western blots, PH did the work associated with electron microscopy, CS has given intellectual input and sorted out the human MND database, CT collected and delivered the human MND samples, TSJ and JWB did the conceptual work on this manuscript including experimental planning and manuscript preparation.

## Supplementary Material

Additional File 1 **APP/Rab7 as a diagnostic marker for impaired vesicle trafficking in MDF motor neurons**. Immunohistochemical staining for Amyloid-Precursor Protein (APP,A) using rabbit anti-APP, and for Rab7 (B) using goat-anti-Rab7, and co-localisation of both proteins (C) in spinal cord motor neurons of 60 d.p.n. MDF mice. Note the large endosomal vesicles (arrows in C) staining positive for APP and Rab7, indicating a transport impairment in these motor neurons, similar to the ones observed in wobbler. Click here for file
