# Supplementary figures and images for: Endosomal accumulation of APP in wobbler motor neurons reflects impaired vesicle trafficking: Implications for human motor neuron disease
Source: BMC Neurosci. 2011 Mar 7;12:24. doi: 10.1186/1471-2202-12-24 (PMC3058068; doi:10.1186/1471-2202-12-24)

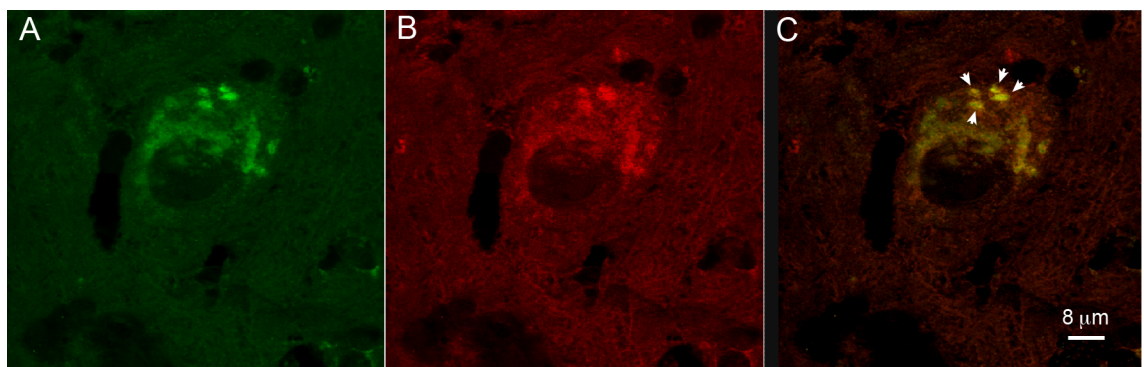

Supplement: Additional File 1 — APP/Rab7 as a diagnostic marker for impaired vesicle trafficking in MDF motor neurons. Immunohistochemical staining for Amyloid-Precursor Protein (APP,A) using rabbit anti-APP, and for Rab7 (B) using goat-anti-Rab7, and co-localisation of both proteins (C) in spinal cord motor neurons of 60 d.p.n. MDF mice. Note the large endosomal vesicles (arrows in C) staining positive for APP and Rab7, indicating a transport impairment in these motor neurons, similar to the ones observed in wobbler. [file 1471-2202-12-24-S1.PDF]
